# Staphylococcal Enterotoxins

**DOI:** 10.3390/toxins2082177

**Published:** 2010-08-18

**Authors:** Irina V. Pinchuk, Ellen J. Beswick, Victor E. Reyes

**Affiliations:** 1 Department of Internal Medicine, University of Texas Medical Branch, Galveston, TX 77555-0655, USA; Email: ivpinchu@utmb.edu; 2 Department of Molecular Genetics & Microbiology, University of New Mexico, Albuquerque, NM 87131, USA; Email: ebeswick@salud.unm.edu; 3 Departments of Pediatrics and Microbiology & Immunology, University of Texas Medical Branch, Galveston, TX 77555-0366, USA

**Keywords:** *Staphylococcus aureus*, enterotoxins, superantigens, class II MHC, food-borne poisoning

## Abstract

*Staphylococcus aureus* (*S. aureus*) is a Gram positive bacterium that is carried by about one third of the general population and is responsible for common and serious diseases. These diseases include food poisoning and toxic shock syndrome, which are caused by exotoxins produced by *S. aureus*. Of the more than 20 *Staphylococcal* enterotoxins, SEA and SEB are the best characterized and are also regarded as superantigens because of their ability to bind to class II MHC molecules on antigen presenting cells and stimulate large populations of T cells that share variable regions on the β chain of the T cell receptor. The result of this massive T cell activation is a cytokine bolus leading to an acute toxic shock. These proteins are highly resistant to denaturation, which allows them to remain intact in contaminated food and trigger disease outbreaks. A recognized problem is the emergence of multi-drug resistant strains of *S. aureus* and these are a concern in the clinical setting as they are a common cause of antibiotic-associated diarrhea in hospitalized patients. In this review, we provide an overview of the current understanding of these proteins.

## 1. Introduction

### 1.1. Source

Staphylococcal enterotoxins are members of a family of more than 20 different staphylococcal and streptococcal exotoxins that are functionally related and share sequence homology. These bacterial proteins are known to be pyrogenic and are connected to significant human diseases that include food poisoning and toxic shock syndrome. These toxins are for the most part produced by *Staphylococcus aureus* (*S. aureus*) although other species have also been shown to be enterotoxigenic. 

*S. aureus* is an ubiquitous Gram-positive coccus of approximately 1 μm in diameter and forms clusters. It colonizes humans as well as domestic animals, and is a common opportunistic pathogen. It is estimated that *S. aureus* is persistent in 20% of the general population, while another 60% are intermittent carriers [1]. Most frequently, the anterior nares is the site of colonization in humans, and this colonization increases the risk of infections when host defenses are compromised. This is supported by multiple observations. For instance, the frequency of infections is higher in carriers than in non-carriers [2]. Non-carriers commonly acquire infections through contaminated food or when food handlers who are carriers contaminate food during preparation.

*S. aureus* is a facultative anaerobe forming yellow colonies on rich medium and causing an α−, β− and double (α + β) hemolysis on blood agar plates [3]. It expresses a wide array of cell-associated and secreted virulence factors. These properties make it a versatile pathogen capable of a wide range of infections. The secreted factors include various enzymes, cytotoxins, exotoxins, and exfoliative toxins. The chief function of these enzymes is to turn host components into nutrients that the bacteria may use for growth. Among the other secreted factors are exotoxins that include staphylococcal enterotoxins (SE), and toxic shock syndrome toxin (TSST)-1 and are the focus of this review. These factors subvert the host immune system and illicit major responses as described below. 

Most genes coding for SEs are located on mobile elements such as plasmids, bacteriophages or pathogenicity islands [4,5]. Thus, horizontal transfer between strains is not rare. In fact, a recent study showed that most *S. aureus* isolates obtained from three separate hospitals had more than one enterotoxin gene [6]. The median number of enterotoxin genes in the *S. aureus* isolates in that study was five and some contained up to 12 enterotoxin genes [6]. Although there are more than 20 distinct staphylococcal enterotoxins, only a few of them have been studied in depth. The most common staphylococcal enterotoxins are SEA and SEB. As shown in Table 1, SEA is the most common toxin in staphylococcus-related food poisoning. SEB, while it is associated with food poisoning, has been studied for potential use as an inhaled bioweapon [7]. SED is suggested to be the the second most common staphylococcal toxin associated with food poisoning worldwide, and one study showed that only very small amounts of this toxin were needed to induce food poisoning [8]. SEE has also been documented in some cases of food poisoning, while SEF has been implicated in toxic shock syndrome [8,9]. SEG, SEH, and SEI are not as well studied as the others, but were associated with one of the food poisoning outbreaks in Taiwan [10]. SEH has been also identified as one of the causes of massive food poisoning associated with the reconstituted milk consumption in Osaka, Japan in 2000 [11]. 

**Table 1:** Unique features of some common SEs.

Staphylococcal Enterotoxin	Feature	Binding to Class II MHC
SEA	Most common toxin associated with staphylococcal food poisoning	Alpha and beta chains [12]
SEB	Studied as a biological weapon	Alpha chain [13]
SEC	Commonly isolated from animals [14]	Outside the binding groove on the flanking helix from the α chain [15]
SED	Food poisoning [16]	Alpha and Beta chains [17]
SEE	Food poisoning [9]	Beta chain [18]
SEF	Associated with toxic shock syndrome [8]	Binds to alpha and beta chains [19]
SEG	Minor role in food poisoning [10]	SEB-like interaction with a chain [20]
SEH	Food poisoning [10,11]	Alpha chain [21]
SEI	Minor role in food poisoning [10]	Beta chain [22]

### 1.2. Structure

Staphylococcal enterotoxins (SEs) are broadly classified as superantigens, which, as described in detail below, have the ability to stimulate large populations of T cells (~20–30%) leading to the production of a cytokine bolus [23,24]. At least 20 serologically distinct staphylococcal superantigens have been described that include SEs A through V and toxic shock syndrome toxin-1 (TSST-1). SEA, SED, and SEE share 70–90% sequence homology, while only 40–60% with SEB, SEC, and TSST-1 [17,24].Their mature length is approximately 220–240 amino acids, depending on the toxin, and their molecular size is on average ~25 kD and have significant sequence variability, but when folded have similar three-dimensional structures [25,26,27]. 

The three dimensional structure for multiple SEs has been determined by crystallography [28,29,30,31,32,33,34,35]. They are by and large elliptical in shape and have two major unequal domains composed mostly of β strands and a few α-helices. The two domains are separated by a shallow cavity. The larger of the two domains contains both the amino and carboxyl termini. Mutational analysis of both SEA and SEB implicated this cavity in the binding to T cell receptors (TcR) [36,37]. Another region on SEA identified by mutational analysis to interact with the TcR Vβ 7 and 8.1 is tyrosine 66, while a stretch of amino acids from 45 to 58 on SEB was found to be involved in the binding to class II major histocompatibility complex (MHC) molecules that are expressed by antigen presenting cells (APC) [38]. Several of these enterotoxins have a Zn-binding site that contributes to their interaction with class II MHC molecules [32,33]. Studies showed that stretch of amino acids (a.a. 118–175) located two thirds of the length of the protein sequence is similar to the COOH-terminal end of the human and mouse CD74 protein (aka invariant chain) [39], which binds class II MHC molecules early during their synthesis in the endoplasmic reticulum and serves as a scaffold for their assembly. The most effective and well studied class II MHC molecule for Staphylococcal enterotoxin binding is the HLA-DR1 allele [40]. HLA-DR has two chains, α and β, that Staphylococcal enterotoxins may bind, some bind to both chains, like SEA and SED, while the others bind one chain or the other as shown in Table 1. SEA has also been examined in binding to other class II MHC isoforms and was shown to successfully bind to HLA-DP and HLA-DQ. SEB and SEC failed to bind to HLA-DP, but did show some interaction with HLA-DQ [41].

### 1.3. Properties

These SE proteins have a remarkable ability to resist heat and acid. Therefore, they may not be completely denatured by mild cooking of contaminated food. They are pyrogenic and share some other important properties that include the ability to induce emesis and gastroenteritis as well as their noted superantigenicity. They are resistant to inactivation by gastrointestinal proteases including pepsin, trypsin, rennin and papain [42]. Thus, they can easily outlast the bacteria that produce them. 

## 2. SEs in Food-Borne Poisoning Associated Diarrhea

A frequently cited, but somewhat dated, estimate by the Centers for Disease Control (CDC) on food-borne diseases is that SEs affect approximately 80 million individuals in the US, alone, resulting in 325,000 hospitalizations and more than 5,000 deaths [43]. According to the World Health Organization, death of about two million individuals in the world is due to food borne diarrheal diseases. The economic impact of food-borne diseases is also substantial. In the US, the estimated costs for these diseases may reach $35 billion annually [44]. 

Staphylococcal food-borne diseases acquired from eating enterotoxin-contaminated food are the second most commonly reported types of food-borne diseases. The high incidence of staphylococcal food poisoning is due to the insufficient pasteurization/decontamination of originally contaminated product source [45] or its contamination during preparation and handling by individuals who are carriers of the organism. Also, since *S. aureus* grows over a wide range of temperatures and pH, the bacteria may grow in a wide assortment of foods. Therefore, food that is contaminated with SE-producing strains, if left at temperatures that allow rapid growth of the bacteria (*i.e.*, inadequate refrigeration) is a common source of SE-outbreaks. 

The amount of toxin needed to cause disease is less than 1 μg. In an outbreak due to enterotoxin (SEA)-contaminated chocolate milk, the amount of toxin was reported to be only 0.5 ng/mL [46]. The disease has a short incubation period that ranges from just a few minutes to hours since the toxin is preformed. Symptoms include nausea, vomiting, abdominal pain, cramps and diarrhea. SEA is responsible for approximately 80% of the cases of food poisoning outbreaks in the USA, while SEB is responsible for 10% of the cases [47,48]. The disease is usually self-resolving, is rarely lethal and the elderly are more susceptible. 

## 3. Staphylococcal Enterotoxins in Nosocomial and Antibiotic-Associated Diarrhea

*S. aureus* is a major cause of nosocomial infections and community-acquired diseases. Diarrhea is a frequent side effect of antibiotic treatment and is prevalent among hospitalized patients, especially those in geriatric wards or intensive care units. The severity of antibiotic associated diarrhea ranges from mild to fatal, such as cases of pseudomembranous colitis. While the causative agent of antibiotic-associated diarrhea is not always easy to determine, *S. aureus* is highly suspected as it can be a member of the gut microflora and stools of antibiotic-associated diarrhea patients have been found to contain enterotoxin-producing strains of *S. aureus* [49]. In one study, investigators examined nosocomial antibiotic-associated diarrhea and found stool specimens that were positive for SEs with a high density of bacteria (10^8^ CFU/g of stool)[50]. It is important to note that most of the antibiotic-associated diarrhea isolates of *S. aureus* are methicillin-resistant (MRSA)[50]. Methicillin is a semisynthetic β-lactamase-insensitive β-lactam. Resistance to this antibiotic is linked to the *mecA* gene that encodes a penicillin-binding protein called PBP2a, which allows the synthesis of the cell wall even at lethal concentrations of β-lactams, because PBP2a has low affinity for β-lactams [51]. MRSA strains are resistant to all β-lactam antibiotics. Thus, MRSA represent the model multi-drug resistant bacterial pathogens. MRSA is a worldwide problem that has increased steadily during the last three decades. In 2003, 60% of *S. aureus* in the ICU were found to be MRSA [52]. Importantly, the majority of the MRSA are toxin producing strains (TSST-1, SEA, SEB, SED [53]). This multi-drug resistant pathogen is among the major concerns in hospitals. How the production of the toxin affect the immunopathogenesis of MRSA associated diarrhea remains unclear. Therefore, the better understanding of the role of *S. aureus*-associated toxins in the immunopathogenesis of MRSA associated nosocomial and antibiotic-associated diarrhea are required to better prevention and treatment of the infection caused by this ancient nemesis.

## 4. Gastro-Intestinal Inflammatory Injury Associated with Enterotoxigenic Diarrheal Diseases

The earlier studies of Gastro-Intestinal (GI) inflammatory injury associated with the SE food poisoning were performed in 1960–1970 using monkey and dog animal models [54,55,56,57,58]. It has been demonstrated that ingestion of SEs within food cause food poisoning, which is characterized by severe vomiting and diarrhea [59], as mentioned earlier. Those symptoms occur within hours after eating of SE-contaminated food [54]. SE food poisoning leads to inflammatory changes throughout the gastrointestinal tract with severe lesions in the jejunum and ileum. The direct inhibitory effect of purified SEs on intestinal tone, contractility and colonic transit has been noted in the dog model [56]. Oral and intraduodenal administration of SEA to weanling pigs was associated with increased numbers of lymphocytes and polymorphonuclear cells in the jejunum and duodenum, quick emetic and neurobehavioral responses [60], suggesting that intestine is a site of SEA action. Intragastric administration of a single dose of SEB to rhesus monkeys produced a lesion confined to the mitochondria in epithelial cells of villi and crypts and was associated with rapid infiltration of leukocytes to lamina propria of jejunum [55]. These changes were concomitant with the evidence of an acute jejunitis. Some early studies deonstrated that the administration of the enterotoxigenic staphyloccoccal extract into the upper ileum through isoperistaltic enterocutaneous feeding fistula resulted in acute ileitis in the dog model [58]. This effect was dose dependent and high doses of SEs resulted in the dilatation, edematous and hyperemic changes in distal ileum. Chronic administration of SE extract resulted in the hypertrophy of mesenteric lymph node and an increase in lymphoid aggregates within the ileal submucosa. Chronic administration of large dose of SE extract was associated with lymphoid hyperplasia in the mucosal lamina propria, submucosal fibrosis and thickening of the bowel wall [56,58]. Mild lymphoid lesions were identified as early as 24 hrs, with severe lymphadenopathy, splenomegaly, and prominent Peyer's patches found at 72 hrs after intravenous SEB administration in the piglet model [61]. Beery *et al*. observed similar inflammatory changes in the rat stomach and duodenum even after administration of single oral dose of SEA and noted predominant intraepithelial lymphocytes responses in jejunum [62]. Moreover, this elegant study showed that the intact rat GI epithelial barrier allowed the prompt passage of orally presented SEA across the epithelium to the lamina propria and, subsequently, to the kidney.

**Figure 1:**
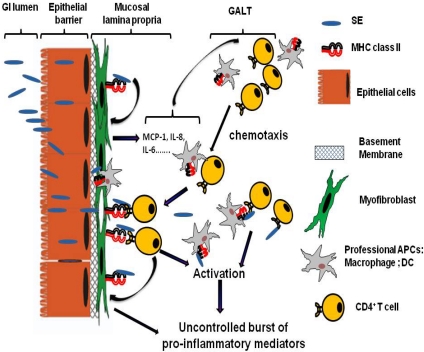
Model of the role of mucosal lamina professional and non professional APCs in SE associated Gastro-Intestinal (GI) inflammatory injury. GI inflammatory injury during staphylococcal enterotoxigenic disease is mediated mostly through the SE superantigenic effect on MHC class II expressing mucosal professional (macrophages and dendritic cells, DC) and non professional (such as myofibroblasts) APCs and TCR expressing CD4^+^ T cells. SE can cross the intestinal epithelial barrier in intact form and bind to class II MHC molecules that expressed on subepithelial myofibroblast. These processes will lead to a strong production of the proinflammatory cytokines and chemokines, including IL-6, IL-8 and MCP-1. The last one may leads to the increased chemotaxis of professional immune cells (CD4^+^ T cells, Macrophages, DC) from gut associated lymphoid tissue (GALT) to the site of SE associated inflammation in GI mucosa. Those MHC class II:SEs:TCR interactions may in turn result in hyperactivation of the APCs and the T cells leading to the excessive proliferation of T cells and the uncontrolled burst of various proinflammatory cytokines and chemokines causing the superantigen-mediated acute inflammation and shock.

Despite the significant progress in the understanding of SE associated inflammation of GI tract, it is still unclear how this inflammation is initiated *in vivo* and what is the exact role of each of the immune and non immune cells that contribute to the progression of the disease. Many recent *ex vivo* and *in vitro* studies suggest that GI inflammatory injury associated with staphylococcal enterotoxigenic disease is mediated mostly through the SE superantigenic effect on MHC class II expressing APCs and CD4^+^ T cells, cells expressing major receptors for SEs [63,64,65,66,67]. Those interactions may result in hyperactivation of professional as well as non-professional APCs and T cells leading to the excessive proliferation of CD4^+^ T cells and the release of proinflammatory cytokines and chemokines that contribute to the SE inflammatory effect on GI tract (Figure 1) [64,65,66,67,68]. 

## 5. Mechanisms of Action

### 5.1. Emetic effect of SEs

Although the superantigenic activity of SEs has been well characterized, as discussed below, the mechanisms behind the emetic activity are poorly understood. In large part, this is due to the dearth of adequate animal models, some of which were mentioned above. Non-human primates represent an ideal candidate, but the high costs and ethical issues prevent their use to study emetic effects of SEs. One animal model that seems well-suited to study the emetic response of SEs is the house musk shrew. This small mammal that resembles a mouse responds with vomiting two hours after peroral or intraperitoneal administration of SEs [69]. Studies by Hu *et al.*, who used the house musk shrew, showed that the small intestine is a site of emetic action by SEA and appears to involve the 5-hydroxytryptamine (5-HT) or serotonin pathway [70]. Serotonin is an important signaling mediator in the gastrointestinal tract and can activate enteric neurons, stimulate muscle responses, and enhance secretion. Their studies showed that SEA-induced emesis was inhibited by cannabinoid (CB) receptor agonists and the action was reversed by a CB1 antagonist [70]. A recent study showed that aspartic acid at position 227 of SEA was important in the emetic activity, since substitution of that amino acid with alanine resulted in a molecule devoid of emetic activity [71]. Histamine and Ca^++^ channel blockers have also been found to prevent the emetic response to SEs suggesting an involvement of mast cells in enterotoxin-induced emesis. 

### 5.2. SE superantigenic property in immunopathogenesis associated with staphylococcal diarrheal disease

**Figure 2:**
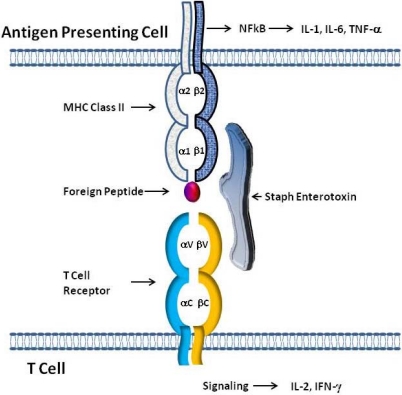
Model of SE interaction with T cell Receptors and class II MHC Molecules. The simultaneous binding of SEs outside of the antigen binding pocket of class II MHC on antigen presenting cells (APC) and to T cell receptors expressing certain Vβ elements allows SEs to act as superantigens. The tripartite interaction of class II MHC:SEs:TcR results in the stimulation of both APC and T cells leading to the production of cytokines by both cell types.

Staphylococcal enterotoxins bind to class II MHC molecules on APCs outside of the antigenic peptide binding groove (Figure 2). The current literature suggests that the binding of these toxins to class II MHC is directed by very few residues, as shown by directed mutagenesis studies with class II MHC [72]. As for TSST-1, mutating a single residue may abolish its binding [73]. SEA has two distinct binding sites on both sides of the peptide binding groove of class II MHC. SEA molecules must be bound to both sites for optimal activity, which allows for class II MHC crosslinking, and stable interactions with T cells [70]. SED was shown to have multiple sites of interaction with class II MHC [17]. SEB and TSST-1 bind to the same region of HLA-DR1, but TSST-1 is the only staphylococcal toxin that extends part way over the peptide binding groove when bound to class II MHC [13]. SEE is similar structurally to SEA and binds to the same region as SEA on the beta-chain [18]. The SEH binding site on class II MHC overlaps with one of the SEA binding sites, and SEI binds to the HLA-DR1 beta-chain [21,22]. Once bound to class II MHC, SEs may then bind to T cells via the T cell receptor (TCR). T cells normally require presentation of a specific antigenic peptide to the TCR by APCs in order to become activated. However, SEs interact with T cells in a “nonspecific” manner, only requiring a common variable region on the TCR (Figure 2). This MHC class II:SEs:TCR tri-molecular interaction leads to an uncontrolled release of various proinflammatory cytokines including IFN-gamma, TNF-α, IL-1β, IL-6 and IL-8, the key cytokines/chemokines causing superantigen-mediated acute inflammation and shock [74,75,76,77]. Whereas T cells are normally only activated in an antigenic specific way, their interaction with SEs leads to a massive proliferation and differentiation of T cells predominantly toward Th1 and Th17 phenotypes [78,79], both of which are associated with acute inflammatory responses. 

### 5.3. Effects on professional and non-professional APCs

The intestinal epithelium is a layer of cells separating the lumen from immune cells, providing a barrier for the vast amount of food antigens, bacteria, and viruses to which the gut is exposed. The extensive inflammation induced by the immune response to SEs leads to an increase in intestinal epithelial permeability and a decrease in expression of tight junction proteins. Disruption of barrier function leads to an influx of antigens through the mucosal layer, further activating immune responses to these antigens as they interact with immune cells. SEs are able to cross the epithelial barrier intact and, by traversing this barrier, gain access to T cells. One study demonstrated that SEB was more efficient at traversing the epithelial barrier than SEA, and thus, is more likely to reach the blood [80]. In addition to inducing T cell responses, SEs also induce proinflammatory responses from professional and non professional APCs when binding to MHC class II on these cells. In a mouse model, SEA, SEB, and TSST-1 were able to induce dendritic cell migration and maturation dependant on T cell activation [81]. Macrophages are also activated by SEs and upon binding release neutrophil chemotactic factors that induce neutrophil migration [82], and increased release of proinflammatory cytokines [83].

Similarly, we have shown that MHC class II-expressing intestinal subepithelial myofibroblasts, non professional APCs in GI mucosa, bind SEs, and are activated to produce proinflammatory cytokines such as IL-6, IL-8, and, to a less extent, TNF-α. Our further studies suggested that SEA was able to cross a monolayer of intestinal epithelial cells and bind to MHC class II expressed on isolated subepithelial myofibroblasts in co-culture, thus, inducing the production of MCP-1 along with the above mentioned proinflammatory cytokines [67]. In these studies, MCP-1 was shown to play an important role in the response to SEA since its neutralization decreased expression of IL-6 and IL-8 during exposure to SEA. Moreover, the results from the same study suggest that SEA induced MCP-1 production by the myofibroblasts might be involved in chemotaxis of lymphocytes to the site of SEA induced inflammation as illustrated on Figure 1. Another cell population that could bind SEs shortly after exposure are intestinal epithelial cells, but their class II MHC expression is prominent mostly during inflammation, and upon homeostasis has been distinctly observed in the duodenum [84,85]. When T cells were added to culture with the epithelial cell exposed to SEs, proliferation was induced suggesting a potential involvement of these cells in SE associated immunopathogenesis. Other studies have shown that interaction of the MHC class II^+^ vascular endothelial cells with SEB, initiates T cell activation [86]. SEA interaction with these endothelial cells described to induce production of IL-8 and TNF-α leading to the endothelial injury [87]. Another study showed that SEA could bind to B cells via MHC class II molecules [88]. Collectively, these studies suggest that SEs may bind to a variety of cell types via MHC class II molecules and these interactions leads to their activation resulting in proinflammatory cytokines and chemokines production and uncontrolled activation of T cells.

## 6. *In Vivo* Modeling of SE Associated Diarrheal Diseases

Despite the knowledge acquired regarding the SE interactions with the host, their effect on immune responses, and a valid animal model of SE associated airway disease, there are only few models of SE-associated diarrheal disease [89,90,91]. Reviews of enterotoxigenic diarrheal disease models include very limited information about the *in vivo* modeling of the SE associated diarrhea [92]. The more susceptible animal species to develop human-like enterotoxigenic disease are non-human primate models, mostly using *Macaca mulatta* [57,93]. When introduced intragastrically, SEA and SEB have been shown to induce emetic responses, diarrhea and GI inflammatory changes in different *Macaca* spp. [55,68,94,95]. Kohrman *et al.* demonstrated that administration of TSS-associated *S. aureus* to baboons resulted in mild symptoms and was associated with decreased food intake and loose stools [96]. Unfortunately, the use of the primate models to study SE diarrheal disease is limited by high costs, short supply, and complexity of animal care.

The dog [56,58,97], pig [60] and piglet [61] models have been successfully used to reproduce some of the features associated with staphylococcal enterotoxigenic disease. These models have clearly demonstrated diarrhea and the appearance of the immunopathological changes in the gut-associated lymphoid tissue (GALT). However, the main disadvantage for use of those models remain similar to the monkey models: high cost and short supply in the available tools for the study of SE associated immunopathology. 

Wild strains of rodents are less susceptible to the SE or TSST-1, as the affinity of those toxins to murine MHC class molecules are much lower [92]. However, one of the first attempts to model SEB associated diarrheal disease was done in rats [98]. This model has been successfully used to study the effects of SEA on the GI tract [62,98]. However, an acceptable murine model to study the immunopathogenesis of SE-associated diarrheal disease has not yet been developed. Recent progress made in the development of “humanized” transgenic animals expressing the human MHC class II alleles, such as HLA-DR and -DQ [99] might be useful to finally get insights in the GI immunopathobiology of SE associated diarrheal disease. 

## 7. Their Potential as Agents of Biological Warfare

SEB is the only known *Staphylococcus* enterotoxin that has been examined as a biological warfare weapon. There was particular interest in weaponizing SEB in the Cold War Era because of its stability and potential simplicity in production and dispersal. SEB was studied in an aerosolized form for use as a weapon. It may be purified from culture supernatants in the laboratory, and therefore would be easy to produce. As mentioned earlier, SEB is quite stable to heat, proteolytic digestion, and a wide pH range [7] also making it easy to produce and distribute. A very small amount (0.004 µg/kg) is effective at inducing symptoms, and a dose of 0.02 µg/kg could be lethal [100]. The fact that a low dose of SEB is sufficient to incapacitate people is another factor that makes it a potential weapon. Inhalation of SEB leads to shortness of breath and chest pain for several hours after exposure. With heavy exposure, more serious symptoms could occur such as high fever, pulmonary edema, possible acute respiratory distress syndrome, or septic shock [101]. Symptoms were examined in both animal studies and in several accidental laboratory accidents. In studies where monkeys were immunized with SEB-containing microspheres, all the monkeys studied developed toxic shock syndrome within 48 hours [102]. There have been several laboratory cases of inhalation that may represent the potential of SEB as a weapon. In the 1960s, three different occurrences of laboratory exposure to SEB were reported under the US Offensive Biological Warfare Program (http://www.cdc.gov/ncidod/EID/vol10no9/04-0250.htm [103]). In 1963, a total of nine people were exposed to aerosolized SEB. Two were exposed due to a ruptured hose in the laboratory containing SEB. Both people suffered fever, headache, gastrointestinal symptoms, but recovered 72 hours later. In a separate incident in 1963, five people of seven exposed became ill while performing experiments with monkeys. The monkeys were being exposed to aerosolized SEB, and it is thought the SEB was carried in the monkey’s fur, exposing laboratory workers while they were handling the monkeys. Within 24 hours of exposure five people experienced fever, cough, chest pain, diarrhea and vomiting. Four of the five people with symptoms were hospitalized, but all survived. The third incident of exposure during this program was in 1964 when a tube of aerosolized SEB meant for monkeys ruptured, resulting in the exposure of fifteen people to SEB. Ten people developed the same symptoms as the previous two laboratory exposures and nine of them were hospitalized. Symptoms were cleared in three-five days.

In addition to inhalation, SEB could be purified and introduced into water or food systems in order to affect large numbers of people. However, the probability of a terrorist having the technical skills to weaponize SEB is low. The more likely scenario is that purified SEB could be stolen from a laboratory so it would more likely be an isolated threat unless large amounts could be stolen. The risk of widespread mortality with the use of SEB as a weapon is low; however, it could effectively incapacitate the general population or soldiers on the front line.

Despite its low mortality threat risk, vaccines against SEB have been examined in the 1960s and after the increased terrorist risks perceived after September 11th, 2001. In the 1960s, the United States Army Medical Research Institute of Infectious Disease (USAMARIID) focused on vaccines containing formalin-inactivated SEB toxin. The objective of the research was to induce protective antibodies in monkeys without major side effects by inactivating the toxin. In the 1960s subcutaneous injection was examined, and thirty years later, intramuscular injection was examined [104,105] with both leading to some protective responses without side effects. However, when the vaccine was administered intranasally it only induced weak responses. Thus, in 2003 research turned to the development of recombinant type of SEB vaccines. The goal for those type of vaccines is to produce a mutated SEB protein, which lacked its toxic property, but remains sufficiently immunogenic to induce protective anti-SEB immune responses [106,107]. 

## 8. Agents that Target the Superantigen Effect of SE

Despite all the advances in the understanding of the SE mechanism of action, the SE-associated diarrheal disease due to food poisoning or nosocomial *S. aureus* infection is of major concern in health programs worldwide. WHO pointed out in 2003 that the best approach to reduce the number of food poisoning-related disease outbreaks are preventative measures and treatments against SEs [108]. The preventive measures include stricter food control, hand and environmental hygiene, identification and isolation of carriers, and proper *S. aureus* antibiotic therapy [109,110,111,112]. 

SE-associated diarrheal disease symptoms are abrupt, and may be severe enough to warrant hospitalization. Although due to the self-limitation of this disease specific anti-staphylococcal therapy is not always required, but it is generally agreed that antimicrobial agents with activity against *S. aureus* should be given to all patients with suspected toxic shock syndrome and MRSA infections [113,114]. However, the increase in MRSA strains poses a challenge to efficient therapy [111,115]. Therefore, novel ways targeting the prevention of SE production by *S. aureus* or blocking/neutralization of SE interaction with the host are required to ameliorate the disease outcome. SE immunopathological effects are strongly associated with their capacity to act as superantigens. Thus, the SE superantigenic properties represent a very attractive therapeutic target. Potential targets to prevent the toxic effects of bacterial superantigens have been well reviewed by Krakauer in 2005 [92], more recently by Fraeser *et al.* in 2008 [116], and Larkin *et al.* in 2009 [117]. Since the discovery of SE structures and immune receptors, multiple immunotherapeutic strategies have been proposed. Those strategies include neutralization of SEs by intravenous Ig therapy that consists of anti-SE polyclonal Abs from multiple donors [118,119,120], blocking the interaction of SEs with MHC class II or TCR [121,122,123,124,125], and inhibition of signal transduction pathways activated by these superantigens, particularly NF-κB [126,127,128]. The inhibition of SE-induced proinflammatory cytokine/chemokine cascade by using neutralizing Abs, anti-inflammatory cytokine (e.g., IL-10), or potent immunosuppressants have been proposed [66,67,129,130]. One study showed that doxycycline treatment inhibited human T cell activation and cytokine release in response to SEs and may have potential as a therapeutic strategy [97]. Mouse studies showed that pirfenidone, rapamycin, and dexamethasone were effective at blocking SEB-induced T cell proliferation and cytokine production [127]. Another recently proposed original approach was to use of the innate immunity modulators [128,131,132]. For instance, Hayworth *et al.* demonstrated that bovine lactoferrin was able to attenuate SEB-induced proliferation, IL-2 production, and CD25 expression in HLA-DR4 transgenic mouse T cells [126]. This inhibition was due to the lactoferrin iron-binding capacity. Dietary plasma protein supplements have been shown to prevent release of SEB-induced mucosal proinflammatory mediators (IFN-γ, TNF-α, IL-6 and LTB4) in rats [132]. 

All the available data has demonstrated that the early blockade of the mechanisms involved in the SE induced hyperactivation of immune responses may represent attractive strategy for the development of new specific anti-SE therapeutic approaches. However, more fundamental *in vivo* studies using adequate animal models are needed to understand, which of those approaches may be the most effective. 

## 9. Concluding Remarks

SEs are members of a large family of bacterial exotoxins produced by staphylococci and streptococci that are functionally and structurally related. They have significant morbidity associated with them and are frequent as *S. aureus* is persistent in 20% of the general population. This population is considered “healthy”. Moreover, *S. aureus* may be transiently carried by as much as 60% of the population. A recognized problem is the increase in MRSA strains, which are dangerous due to their resistance of most antibiotics used in clinical practice. Additional work is needed to develop improved preventive and therapeutic strategies targeting neutralization or impairment of SE induced hyperactivation of the proinflammatory immune responses. 
